# Preparation, purification, and biochemical of fat-degrading bacterial enzymes from pig carcass compost and its application

**DOI:** 10.1186/s12896-023-00818-1

**Published:** 2023-11-03

**Authors:** Xinran Duan, Wei Zhai, Xintian Li, Sicheng Wu, Ye Wang, Lixia Wang, Wangdui Basang, Yanbin Zhu, Yunhang Gao

**Affiliations:** 1 College of Veterinary Medicine, Jilin Agricultural University, Changchun, 130118 China; 2 Northeast Institute of Geography and Agroecology, Chinese Academy of Sciences, Changchun, 130102 China; 3 Institute of Animal Husbandry and Veterinary Medicine, Tibet Academy of Agricultural and Animal Husbandry Science, Lhasa, 850009 China

**Keywords:** *Bacillus* sp., Lipase, Optimization, Purification, Characterization

## Abstract

**Background:**

A lot of kitchen waste oil is produced every day worldwide, leading to serious environmental pollution. As one of the environmental protection methods, microorganisms are widely used treating of various wastes. Lipase, as one of the cleaning agents can effectively degrade kitchen waste oil. The composting process of pig carcasses produces many lipase producing microorganisms, rendering compost products an excellent source for isolating lipase producing microorganisms. To our knowledge, there are no reports isolating of lipase producing strains from the high temperature phase of pig carcass compost.

**Methodology:**

Lipase producing strains were isolated using a triglyceride medium and identified by 16S rRNA gene sequencing. The optimal fermentation conditions for maximum lipase yield were gradually optimized by single-factor tests. The extracellular lipase was purified by ammonium sulfate precipitation and Sephadex G-75 gel isolation chromatography. Amino acid sequence analysis, structure prediction, and molecular docking of the purified protein were performed. The pure lipase's enzymatic properties and application potential were evaluated by characterizing its biochemical properties.

**Results:**

In this study, a lipase producing strain of *Bacillus* sp. ZF2 was isolated from pig carcass compost products, the optimal fermentation conditions of lipase: sucrose 3 g/L, ammonium sulfate 7 g/L, Mn^2+^ 1.0 mmol/L, initial pH 6, inoculum 5%, temperature 25 ℃, and fermentation time 48 h. After purification, the specific activity of the purified lipase reached 317.59 U/mg, a 9.78-fold improvement. Lipase had the highest similarity to the GH family 46 chitosanase and molecular docking showed that lipase binds to fat via two hydrogen bonds at Gln146 (A) and Glu203 (A). Under different conditions (temperature, metal ions, organic solvents, and surfactants), lipase can maintain enzymatic activity. Under different types of kitchen oils, lipase has low activity only for ‘chicken oil’, in treating other substrates, the enzyme activity can exceed 50%.

**Conclusions:**

This study reveals the potential of lipase for waste oil removal, and future research will be devoted to the application of lipase.

**Supplementary Information:**

The online version contains supplementary material available at 10.1186/s12896-023-00818-1.

## Background

Around the world, large quantities of used cooking oil are generated every day [1], used cooking oil is mainly composed of animal and vegetable fats and oils. Waste oil pollution has been reported in different countries, the United States and China generate the most kitchen waste oil, 55,000 tons and 50,000 tons respectively [2], and Malaysia can produce 2–5 kg of waste oil per household [3]. When waste oil enters the environment, it causes clogging and corrosion of sewer pipes, damages water quality, oxidizes in the air, produces foul-smelling gases, causing serious environmental pollution. Waste oil can harm the human nervous system and digestive system [4]. Waste oil entering water bodies can lead to local water eutrophication, causing microorganisms, phytoplankton, algae to grow wildly, and disrupt the original ecological balance [5]. Waste oil is usually mixed with kitchen waste for landfills or incinerated [6]. However, when treated, large amounts of greenhouse gases are released, thus causing secondary pollution [7]. To solve this problem, microbial treatment is proposed. Microbial treatment is an environment friendly and promising method. Lipase from microorganisms can be used as a flexible biocatalyst in different industries such as detergents, oleochemicals, food additives, biodegradable polymers, biodiesel, and textiles [8], and lipase can accelerate the breakdown of triacylglycerols into glycerol and free fatty acids [9]. Different lipase producing bacteria were isolated from different sources, a lipase producing strain was isolated from Irish mountain soil, identified as *Pseudomonas brenneri*, and the produced lipase can be used to synthesize fatty acid methyl esters [10]. Ritika Joshi et al. prepared lipase with *Fusarium incarnatum* KU377454, and after optimization, lipase activity could reach 38.35 U/ml. A rod-shaped *Bacillus amylolyticus* was isolated from soil, and the lipase production could reach 80.63 U/ml when incubated at 40 ℃, pH 7.0, and 3–5% inoculum for 72 h. Mandari et al. isolated *Aspergillus niger* MTCC 872 with 269.87 ± 8.09 U/ml lipase activity at 35 ℃, pH 7, and 70% initial water content [11]. The composting process of pig carcasses produces many lipase producing microorganisms, making the compost product an excellent source for isolating lipase producing microorganisms [12]. In addition, the temperature change of compost follows a typical three-stage pattern: warming, high temperature, and cooling [13]. The high temperature period can reach above 55 ℃, and the strains isolated during this period will show good heat resistance [14]. To our knowledge, no lipase producing strains have been isolated from pig compost.

In this study, a lipase producing bacterium was isolated from the high temperature composting stage of pig carcasses and identified as *Bacillus* sp.. It was purified by optimizing its enzyme production conditions. Next, the purified lipase was subjected to identification, structure prediction, and molecular docking as well as a series of biochemical characterization experiments, and the enzyme properties, thermal stability, and enzyme activity of the lipase were analyzed in the treatment of different animal and plant fats and oils.

## Materials and methods

### Reagents and mediums

Triglyceride medium: peptone 10 g/L, yeast extract 5 g/L, NaCl 5 g/L, triglyceride 2 mL/L, and agar 20 g/L. LB medium: Peptone 10 g/L, yeast extract 5 g/L, NaCl 10 g/L, inorganic salt medium (MSM): CH_3_COONa-3H_2_O 1.0 g/L, NH_4_Cl 1.0 g/L, NaCl 1.0 g/L, KH_2_PO_4_ 0.5 g/L, K_2_HPO_4_ 1.5 g/L, and MgSO_4_·7H_2_O 0.2 g/L; pH 7 ± 0.2, the solid medium was made by the addition of 2% agar powder, all the above reagents are analytically pure and purchased from Aladdin Biochemical Technology Co (Shanghai, China). Citric acid-disodium hydrogen phosphate buffer (20mM, pH = 7.0), polyethylene glycol 20,000 were purchased from Sangon Biotech (Shanghai, China), Sephadex G-75 column were purchased from Macklin (Shanghai, China).

### Morphology and identification of lipase producing bacterium

As a source of microorganisms, samples were collected from pig carcasses and sawdust compost during the high temperature period on day 10 (when temperatures were as high as 56.3 ℃) and transported immediately to the laboratory. The compost sample information (the diseased pig carcasses used in the experiment came from farms around Jilin Agricultural University) is shown in Zhai [15]. About 10 g of sample was added to a 150 mL conical flask containing 90 mL of sterilized 0.85% saline and glass beads. The conical flask was shaken at 37 ℃ and 120 rpm for 2 h, then left to stand for 30 min. 100 μL of supernatant was taken and inoculated in glycerol tributyrate medium (peptone 10 g/L, yeast extract 5 g/L, NaCl 5 g/L, tributyl glyceride 2 ml/L and agar 20.00 g/L). Incubate at 37 ℃ for 48 h. The strain with the largest ratio of the diameter of the hydrolyzed circle to the diameter of the strain was selected as the best lipase producing strain [16], which was purified on Luria Bertani (LB) medium and evaluated by gram staining and microscopic observation. The bacterial genome was extracted using a DNA extraction kit (Sangon Biotech, Shanghai, China). The bacterial genome was used as a template, and 27F and 1492R were used as primers for 16S rRNA gene amplification. The PCR reaction system: template 1 μL (100 μg/μL), 27F 1 μL (10 μM), 1492R 1 μL (10 μM), PCR master mix 12.5 μL, ddH_2_O 9.5 μL. The final concentration of PCR reaction reagent was 0.4 μM. Finally, the PCR products were submitted to Sangon Biotech (Shanghai, China) for nucleotide sequencing, the sequencing results were compared with the 16S rRNA sequences on NCBI by BLAST. Nucleotide sequences of strains close to their homology were obtained from GenBank, and the sequence of ZF2 was repeatedly compared with other sequences using the Clustal W comparison method. Software MEGA 11.0. was used to build a phylogenetic tree by neighbor-joining (NJ) methods.

### Determination of lipase activity and protein concentration

Individual colony was inoculated in 5 mL MSM, cultured in a shaker at 37 ℃, 120 rpm until the OD_600_ reached 1.0. 1% (v/v) cultures were inoculated into a 150 mL conical flask containing 100 mL MSM and incubated at 37 ℃, 120 rpm for 48 h. The cultures were centrifuged (12,000 rpm, 4 ℃, 20 min) and the supernatant was collected as crude lipase. Lipase activity was defined as the substrate hydrolysis in 1 min produces 1 μmol titratable fatty acid, which is 1 U/g (U/mL) [17]. In this study, lipase activity was determined titrimetrically at pH 9 and 55 ℃ using olive oil emulsion as a substrate. Protein concentrations were determined by the BCA Protein Assay Kit (Thermo Fisher Scientific, Rockford, USA).

### Lipase yield optimization

To optimize the production of lipase, the medium composition and culture conditions were gradually optimized. In the optimization experiment, each treatment group was replicated three times, and all of them were incubated for 2 d according to the conditions of 1% inoculum, 37 ℃, and 120 r/min.

### Optimal carbon source, nitrogen source, and metal ions

Sodium citrate, sucrose, glucose, maltose, and sodium carboxymethyl cellulose were used to replace CH_3_COONa in the fermentation medium as carbon sources, respectively, and the enzyme activities were determined. After the optimal carbon source selection is completed, the carbon source concentration was adjusted to 1, 3, 5, 7, and 9 g/L, and the enzyme activities were measured to determine the optimal carbon source concentration. NH_4_Cl in the medium was replaced with (NH_4_)_2_SO_4_, NaNO_3_, urea, peptone, and yeast extract as the nitrogen source, and the enzyme activities were determined. After determining the optimal nitrogen source, the nitrogen source was adjusted to 1, 3, 5, 7, and 9 g/L, and the enzyme activity was measured to determine the optimal nitrogen source concentration. In addition to the optimal carbon and nitrogen sources, Cu^2+^, Mn^2+^, Mg^2+^, Zn^2+^, Fe^3+^, Fe^2+^, and Ca^2+^ were added at a 1 mmol/L final concentration to determine the enzyme activities. After selecting the optimal ion, ion concentrations were adjusted to 0.5, 1.0, 1.5, 2.0, and 2.5 mmol/L to determine enzyme activities, based on enzyme activity, the optimal concentration was selected.

### Optimal culture conditions

Based on the optimized fermentation medium, the culture was carried out according to the initial pH of 4, 5, 6, 7, 8, and 9, the inoculum amount of 1%, 3%, 5%, 7%, and 9%, the incubation temperatures of 20, 25, 30, 35 and 40 ℃, and the incubation times of 12, 24, 36, 48, 60 and 72 h, respectively. Subsequently, the enzyme activities were measured and the optimal culture conditions were selected according to the enzyme activities.

### Lipase purification

#### Ammonium sulfate graded precipitation

20 mL of the crude enzyme solution was added to eight 50 ml beakers, respectively, and solid powdered ammonium sulfate was added under ice bath conditions and stirred until saturation was 10, 20, 30, 40, 50, 60, 70, and 80%, respectively. Refrigerate at 4 ℃ overnight. Then centrifuge at 12,000 r/min for 25 min at 4 ℃. The precipitate was redissolved with 10 mL citric acid-disodium hydrogen phosphate buffer and dialyzed by using a dialysis bag (14 KDa). The dialysate was changed every 4 h and tested with BaCl_2_ until no BaSO_4_ was produced. The solution was concentrated with polyethylene glycol 20,000 and filtered through a 0.22 filter membrane. Finally, the lipase activity and protein concentration in the supernatant and precipitate were measured to select the optimal ammonium sulfate salting interval. Subsequent experiments were performed with the optimal ammonium sulfate salting interval.

### Sephadex G-75 gel exclusion chromatography

The lipase was further purified by Sephadex G-75 gel exclusion chromatography. The column was pre-equilibrated with citric acid-sodium hydrogen phosphate buffer and detected by a 280 nm UV detector. When the value stopped fluctuating, equilibration was completed. The elution flow rate was adjusted to 0.5 mL/min, and one tube was collected every 3 mL, the enzyme activity was measured in each tube. Finally, molecular weights were estimated by SDS-PAGE, proteins were separated with 12% (W/V) acrylamide, and molecular weight was assessed with the protein molecular mass marker (Thermo Fisher, Shanghai, China). Gels were stained with 0.25% Komas blue (R-250) and decolorized in 1% acetic acid.

### Amino acid sequencing, homology modeling and molecular docking

The tape samples were prepared and lyophilized, and 40 μl of trypsin buffer was added and incubated at 37 °C for 16-18 h. The mobile phase A was 0.1% formic acid aqueous solution and B was 0.1% formic acid acetonitrile aqueous solution. The liquid chromatography column (0.15 mm*150 mm, RP-C18, Column Technology Inc) was equilibrated with 95% A solution, the samples were loaded by an autosampler into Zorbax 300SB-C18 peptide traps (Agilent Technologies, Wilmington, DE), and separated in a liquid chromatography column with a liquid phase gradient: 0–15 min, linear gradient from 4 to 50% for B solution; 15–19 min, linear gradient from 50 to 100% for B solution; 19–20 min, liquid B was maintained at 100%. Subsequent mass spectrometry was performed using a mass spectrometer Q Exactive, analysis time: 20 min, detection: positive ions. The software MaxQuant 1.5.5.1 was used to search the corresponding database for raw files of mass spectrometry assays, and the protein identification and quantitative analysis results were obtained. Based on the identification results, primary structure analysis and secondary and tertiary structure prediction, homology modeling, and crystal structure analysis were performed on the proteins.

### Effect of different factors on lipase activity

To investigate the effect of different factors (including heat stability, metal ions, organic solvents, and surfactants) on lipase activity, the enzyme activity of the control group was set to 100%. The experimental groups of lipases were incubated at 30–80 °C, 1 mM and 5 mM Ag^+^, K^+^, Li^+^, Co^+^, Ca^2+^, Cu^2+^, Mg^2+^, Mn^2+^, Fe^2+^, Zn^2+^ and Fe^3+^, 5% and 10% ethanol, isopropanol, DMSO, methanol, n-hexane, and toluene, SDS, Tween 20, Tween 80, gum arabic, and Triton-x-100, respectively. The heat stability was also examined and enzyme activity was determined.

### Catalytic activity of lipase for different substrates

To study the action of lipase on different substrates, the substrates were replaced by animal fats (lard, chicken oil, and butter) and vegetable fats (sesame oil, soybean oil, corn germ oil, peanut oil, and linseed oil). The enzyme activity was defined as 100% when olive oil was used as the substrate, and this was used as a basis to determine the lipase activity with other substrates. All animal and vegetable fats and oils are commercial kitchen oils.

### Data analysis

The resultant graphs were plotted using GraphPad Prism 9.0 and statistical analyses were performed using SPSS 22.0, with one-way ANOVA selected for significance analysis. Data points are means of three experiments and error bars represent standard errors. (* (*p* < 0.05); ** (*p* < 0.01). 0.01 *** (*p* < 0.001)).

## Results and discussion

### Morphology and identification of lipase producing strains

**Fig. 1:**
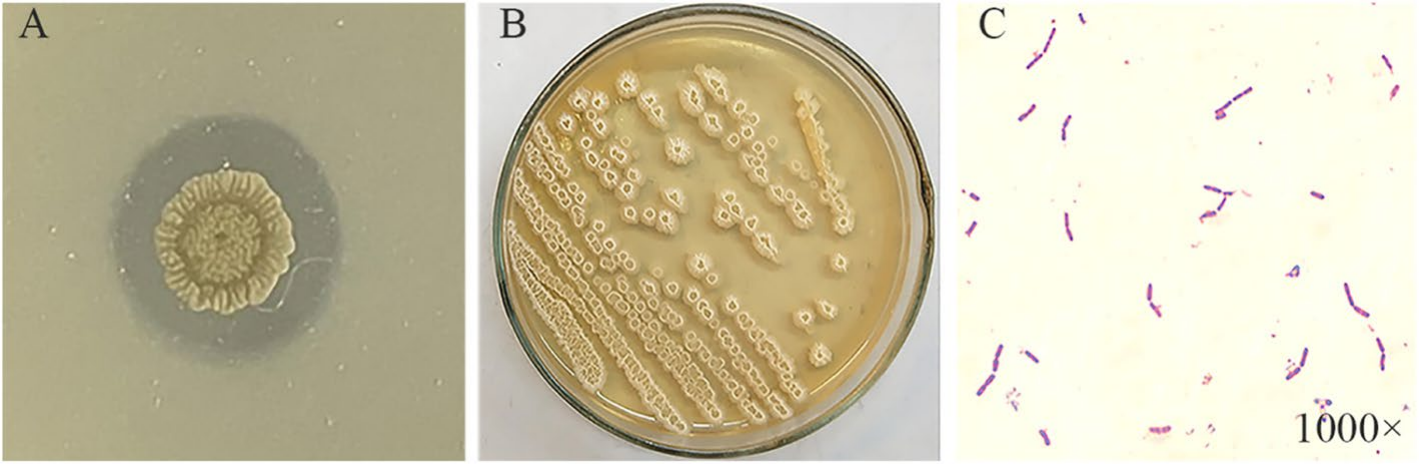
Trisbutyrate glycerol ester hydrolysis circle (**A**), colony (**B**) and cell morphology (**C**) of strain ZF2

A total of four lipase producing and non-pathogenic strains were isolated from compost. Among all strains, strain ZF2 had the largest ratio of hydrolysis circle diameter to strain diameter of 1.69 (Fig. 1A, and Table S1), indicating that it had the highest extracellular lipase activity. Therefore, strain ZF2 was selected for subsequent experiments. On LB medium, the colonies of strain ZF2 showed rough irregular edges with white centers and yellowish edges (Fig. 1B). Gram staining was positive and the bacterium was rod-shaped (Fig. 1C).

**Fig. 2:**
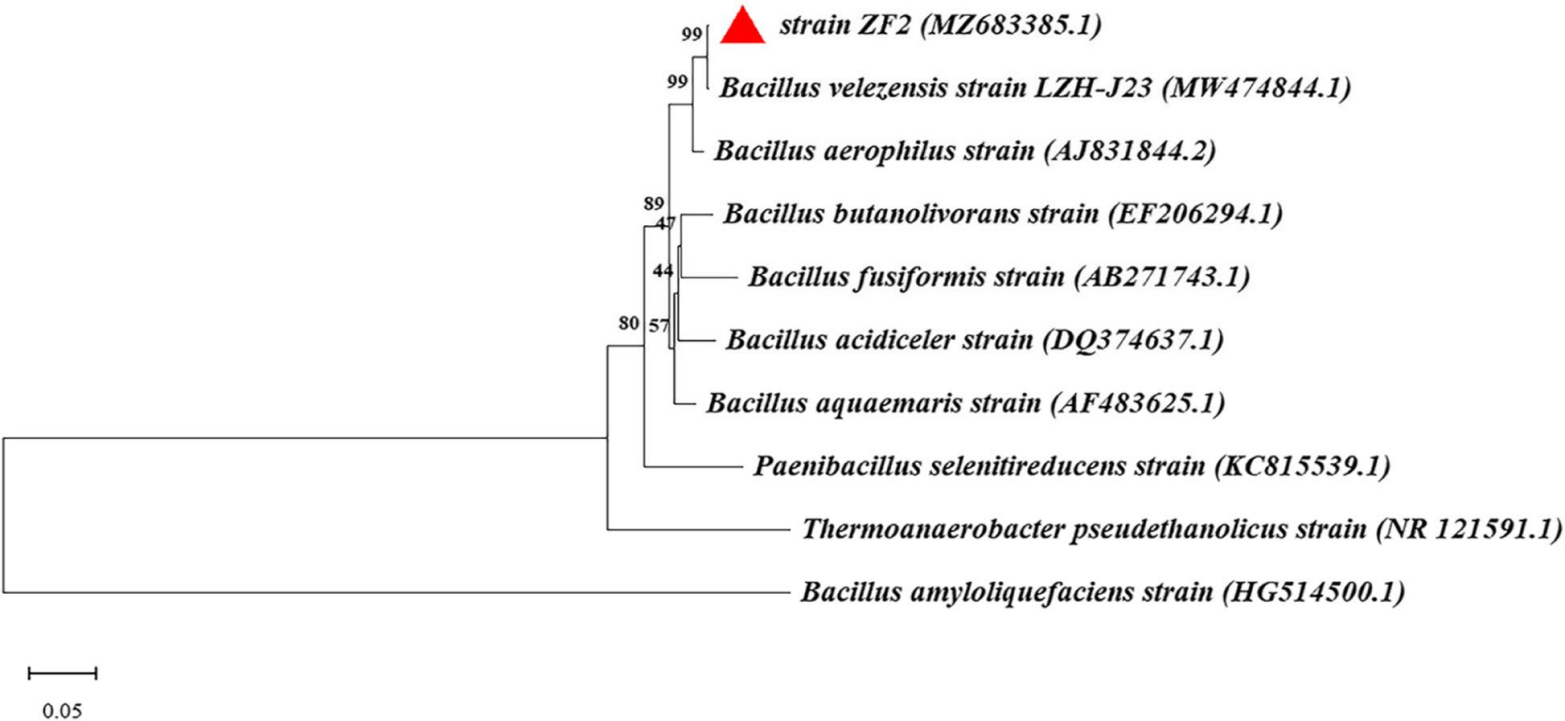
Phylogenetic analysis of strain ZF2

The 16S rRNA gene sequence of the bacterium has been uploaded to the GenBank database with the accession number MZ683385. Eight strains with the closest affinity were randomly selected to construct an evolutionary tree, and strain ZF2 was found to be 98.00% sequence similarity to *Bacillus velezensis* strain LZH-Z23 (Fig. 2). Therefore, strain ZF2 was defined as *Bacillus* sp.. *Bacillus* showed good bioremediation potential in degrading fats [18], oils, and odor reduction [19]. The reports on lipase producing *Bacillus* are less, so follow-up studies were conducted.

### Optimal carbon source, nitrogen source, and metal ions for lipase production

**Fig. 3:**
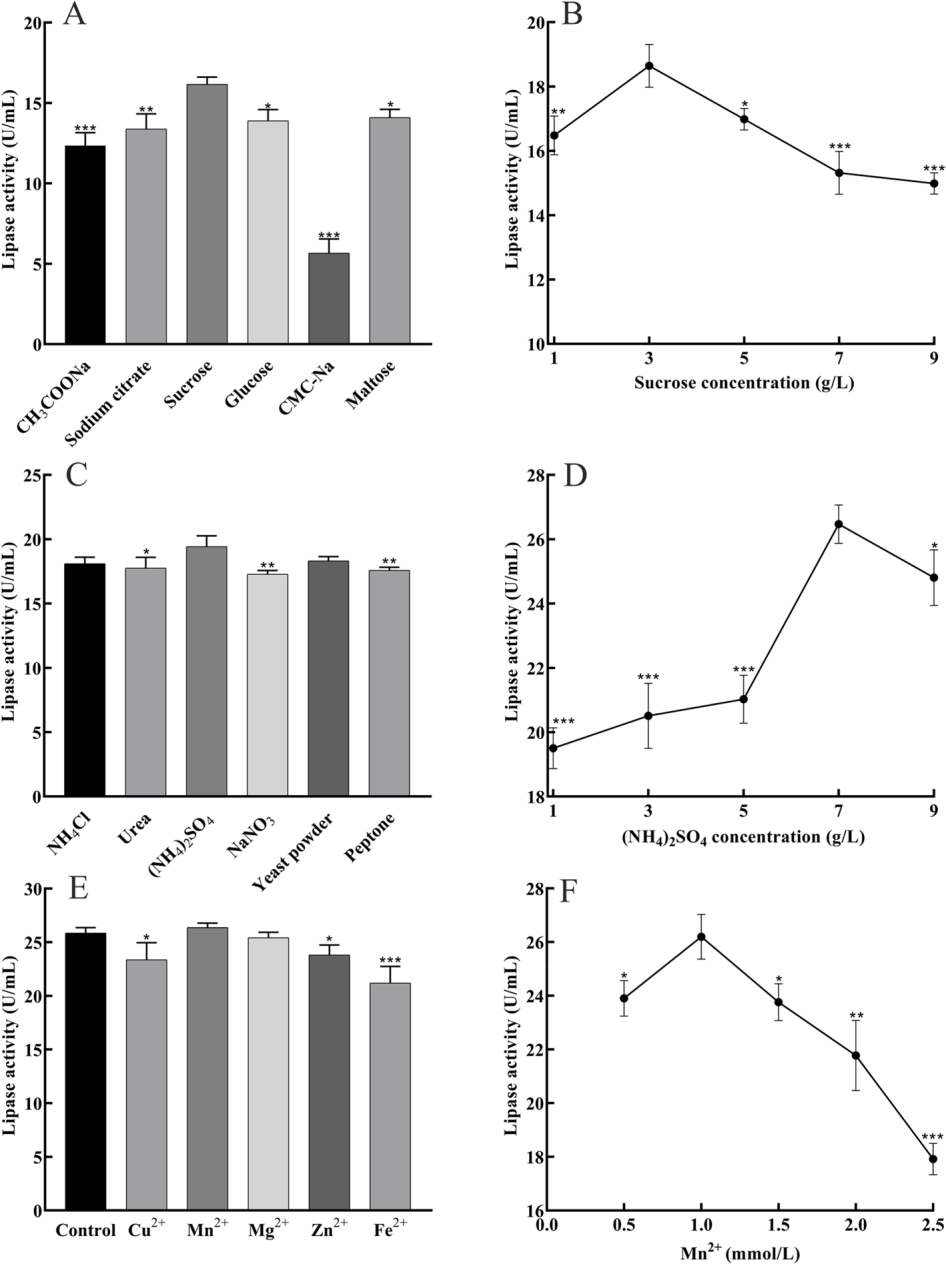
Changes in lipase production under different medium compositions. **A** Carbon source; **B** Sucrose concentration; **C** Nitrogen source; **D** (NH4)2SO4 concentration; **E** Metal ions; **F** Mn2 + concentration

Different culture conditions favor biochemical reactions and increase enzyme activity of the strain [20]. In this section the enzyme production conditions of strain ZF2 were optimized. Carbon and nitrogen sources are the energy sources for bacterial growth [21]. We investigated the effect of different medium compositions on the lipase activity of strain ZF2 (Fig. 3). It could be noted that among the selected carbon sources, sodium citrate, sucrose, glucose, and maltose all promoted lipase activity, with sucrose having the strongest promotion effect, with enzyme activity reaching 16.67 U/mL (Fig. 3A). Enzyme activities were lower under the remaining carbon source conditions than under sucrose conditions (p < 0.05). Lipase activity reached its maximum value (19.31 U/mL) with the addition of (NH_4_)_2_SO_4_ under different nitrogen sources (Fig. 3C). Whereas under the influence of different metal ions, only the addition of Mn2 + increased the lipase activity up to 26.19 U/mL (Fig. 3E), all other metal ions decreased the lipase activity(p < 0.05). Different substances have different effects on the enhancement of enzyme activity, which may be due to different mechanisms.

After identifying the carbon source, nitrogen source, and metal ions, the optimal concentrations were further explored. The enzyme activity increased gradually with increasing substrate concentration, and the optimal concentrations of sucrose, (NH_4_)_2_SO_4_, and Mn^2+^ were 3 g/L, 7 g/L, and 1.0 mmol/L, respectively, under which the enzyme activity could reach 18.64 U/mL, 26.31 U/mL, and 26.19 U/mL, respectively (Fig. 3B, D, and F). As the concentration continues to increase, there is an inhibitory effect on enzyme activity, indicating that the substrate has an optimal concentration of action.

### Optimal inoculum volume, pH, temperature, and incubation time for lipase production

**Fig. 4:**
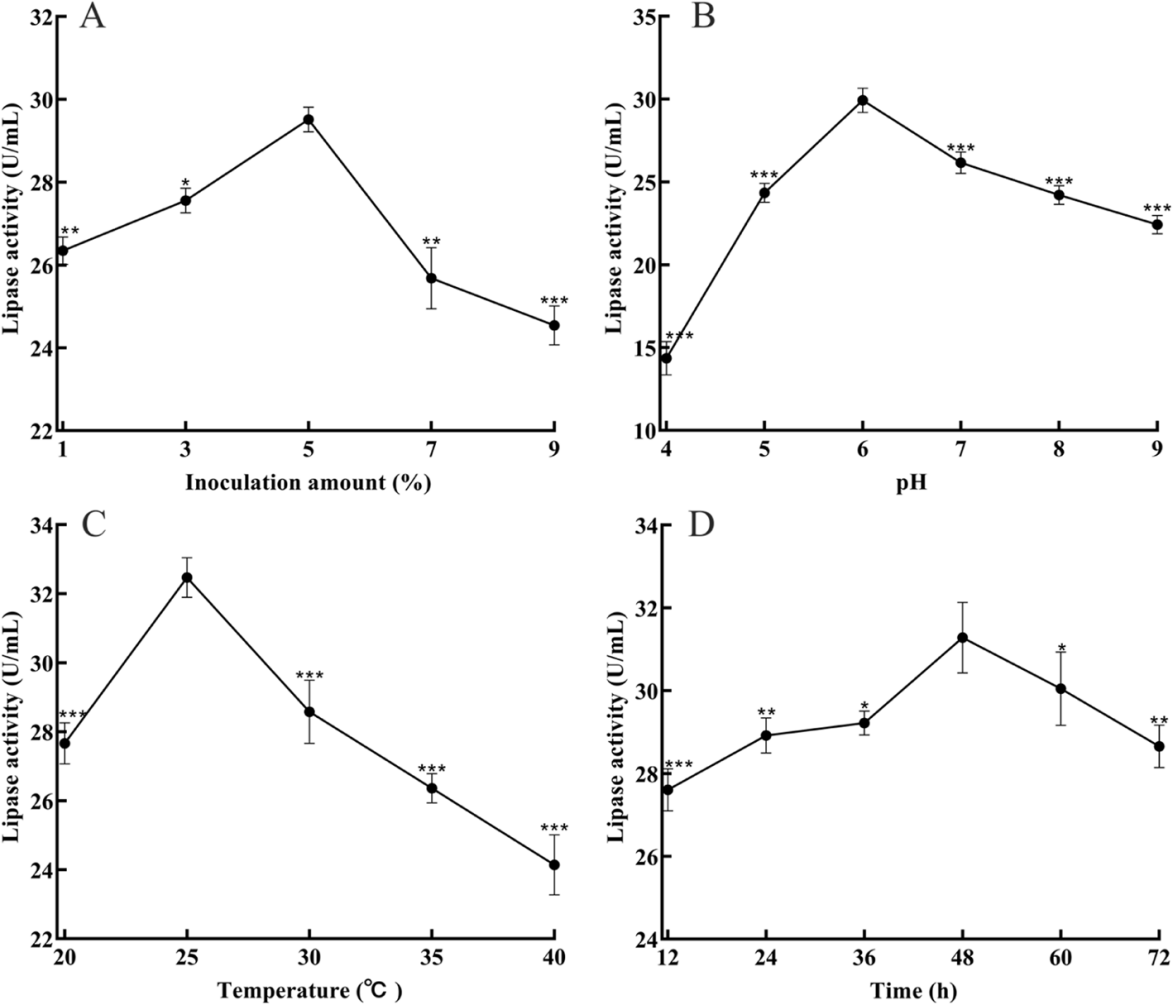
Changes in lipase production under different culture conditions. **A** Inoculation amount; **B** Initial pH value; **C** Temperature; **D** Fermentation time

The appropriate inoculum volume, pH, temperature, and incubation time can maximize the enzyme production capacity of the bacteria. Based on this, the optimization of culture conditions was carried out. The effect of different culture conditions on ZF2 lipase activity is shown in Fig. 4. The enzyme activity increased with the increase of inoculum, at 5% inoculum, the maximum lipase activity was reached (29.51 U/mL), and with the inoculum further increased (*p* < 0.01), the lipase activity decreased, so the optimal inoculum was determined to be 5% (Fig. 4A). Similar results exist in other studies, for *Pseudomonas stutzeri* MTCC 5618, at 1% inoculum, the lipase activity increased by 83.33%, as the inoculum volume increased from 3 to 6%, the enzyme activity decreased [11]. This indicates that the right inoculum volume, nutrient, and oxygen levels are suitable for bacterial growth, thus maximizing lipase production.

At different pH, the lipase activity tended to increase and decrease. At pH 6, the highest enzyme activity (29.92 U/mL) was achieved (Fig. 4B). The effect of temperature on lipase activity is shown in Fig. 4C. In the range of 20–40 ℃, 25 ℃ was the optimum temperature, and lipase activity could reach 31.97 U/mL. In the previous study, the enzyme activity of *Bacillus subtilis* BSN314 was highest at 35 ℃ and pH 7.5. The enzyme activity was affected by high or low temperature and pH [22]. High temperature, low temperature or high, low pH inhibited bacterial metabolism, which may be the reason for the decrease in enzyme activity.

In general, extracellular lipases are produced during the logarithmic late or stable phase of microbial culture [23]. The effect of incubation time on enzyme activity is shown in Fig. 4D. As fermentation proceeded, lipase production gradually increased and reached the maximum lipase activity (32.46 U/mL) at 48 h. As the incubation time was prolonged, the lipase activity started to decrease (p < 0.05), possibly due to bacterial growth gradually slowing down. Therefore, the optimal incubation time is 48 h. The fermentation time of *Pseudomonas putida* 922 was 48 h [24]; the optimal fermentation time of *Pseudomonas aeruginosa* JCM5962 was 4 days [25], the different fermentation times may be related to the different characteristics of the bacteria. By optimizing the medium composition and culture conditions, the optimal conditions: sucrose 3 g/L, ammonium sulfate 7 g/L, Mn^2+^ 1.0 mmol/L, inoculum volume 5%, initial pH 6, temperature 25 ℃, and fermentation time 48 h.

### Purification of lipase

**Fig. 5:**
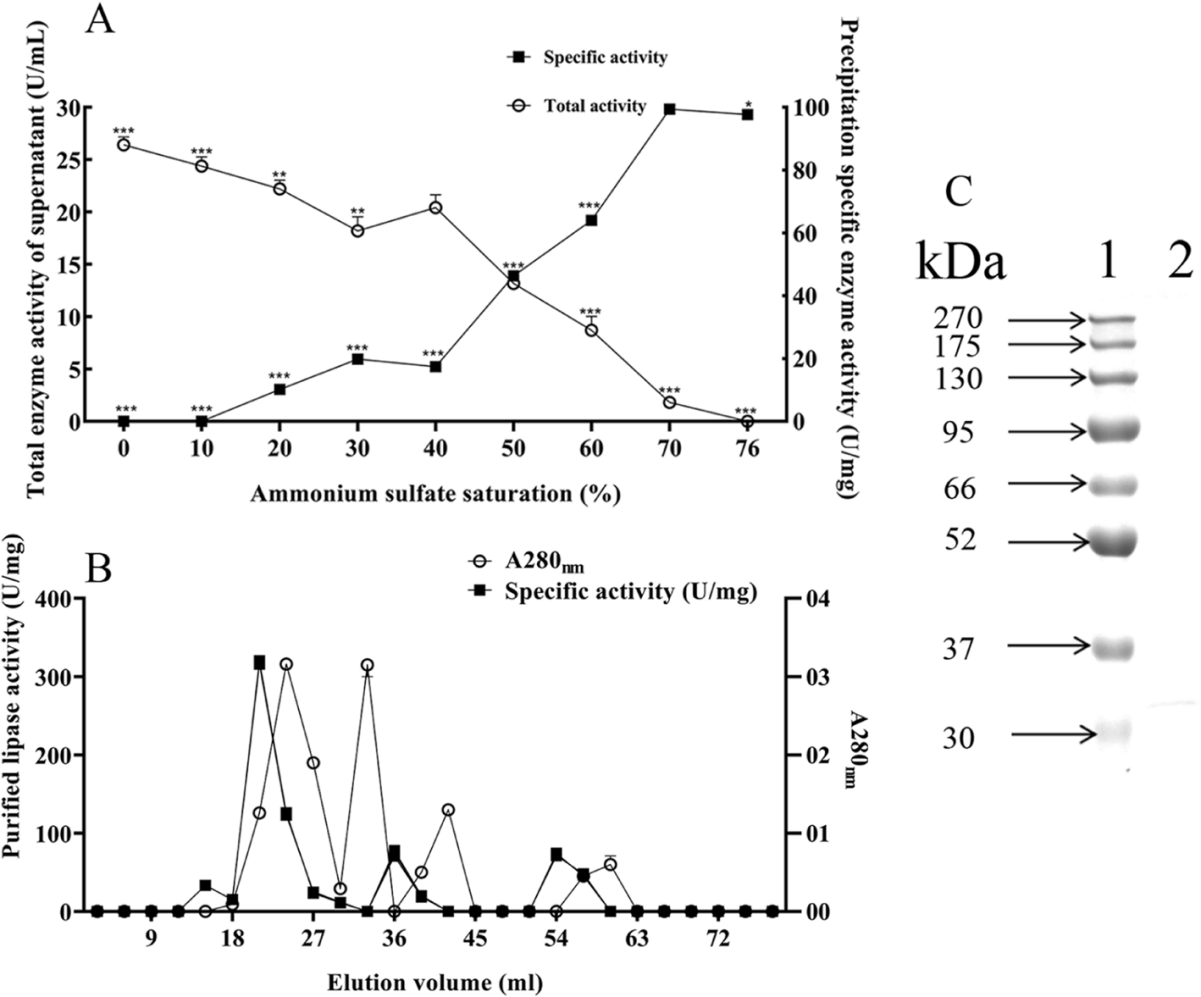
Purification of lipase. **A** Graded precipitation of ammonium sulfate; **B** Sephadex G-75 gel exclusion chromatography; **C** SDS-PAGE images of purified lipase. Lane 1: Marker; Lane 2: Sephadex G-75 purified lipase

The results of the lipase purification are shown in Fig. 5. As the ammonium sulfate increased, the lipase activity in the supernatant gradually decreased. Meanwhile, the specific activity of lipase in the precipitate gradually increased, indicating a gradual transfer of lipase from the supernatant to the precipitate. As ammonium sulfate saturation increased, the specific activity of lipase in the precipitation substantially increased, and the maximum enzyme activity was achieved when the ammonium sulfate saturation reached 70%. However, the specific activity of the lipase in the precipitation decreased when the saturation of ammonium sulfate increased from 70 to 76% (*p* < 0.05), indicating that after the lipase was completely precipitated, the precipitated of other proteins affected the enzyme activity. Therefore, 40% to 70% ammonium sulfate saturation was used to precipitate the crude lipase (Fig. 5A).

The lipase was further purified by Sephadex G-75 gel filtration chromatography. As shown in Fig. 5B, four protein peaks were observed during elution from the Sephadex G-75 gel column and the highest specific lipase activity was detected in the second peak (18–33 mL). Therefore, this peak was chosen as the target peak of the purification process. After two purification steps, the specific activity of lipase increased from 32.46 to 317.59 U/mg, which was 9.78-fold higher than that before purification. The SDS-PAGE image of the purified lipase is shown in Fig. 5C.

### Identification of purified lipase

Based on the LC–MS/MS analysis of lipase, 33 proteins and 52 peptides were identified. Among the obtained proteins, the information for the proteins scoring over 10 is shown in Table 1. Since the protein strips cut after SDS-PAGE gel separation contained multiple proteins, the protein with the highest score was selected for analysis: the highest protein score was 206.51 with a theoretical molecular weight of 27.419 kDa. Secondary mass spectra are shown in Fig. S1A. A total of 240 amino acids from the amino acid sequences were analyzed using BioXM software, and the results showed that a total of six classes of amino acids were included: aliphatic amino acids, aromatic amino acids, sulfur-containing amino acids, basic amino acids, acidic amino acids, and alcohol hydroxyl amino acids (Table S2). The amino acid sequences were subsequently subjected to BLAST and functional analysis in the UniProt protein database (https://www.uniprot.org). The results showed that the lipase has 90.4% homology with the extracellular chitosanase produced by *Bacillus subtilis* 168 (Entry O07921) (Fig. S2). The function of chitosanase includes anti-bacterial, anti-fungal properties, and organism regulation [26], this shows the potential function of lipase.

**Table 1:** Partial LC–MS/MS Matching Protein Information of Lipase

Entry	Protein names	Peptides 1	Mol. weight [kDa]	Score	Sequence coverage1[%]
A0A4Y5UXT4	GH family 46 chitosanase	15	27.419	206.51	36.4
P00782	Subtilisin BPN	5	39.181	32.246	13.9
S6FYV7	Malate deh Ydrogenase	1	33.567	17.625	3.8
A0A8F6D4X3	ABC transporter permease	1	42.3461	13.244	1.8
A0A4V7TMJ9	Ornithine carbamoyltransferase	2	34.517	12.3	6.3

The molecular chemical formula, isoelectric point, and molecular mass of the protein were analyzed using ProtParam software (https://web.expasy.org/protparam), the results are shown in Table 2. The protein has 240 amino acids, the molecular weight is 27,419.43 Da, the molecular formula is C_1199_H_1875_N_341_O_387_S_5_, and the theoretical isoelectric point is 5.44. The instability index (II) of the protein is 39.63 less than 40, indicating that it is a stable protein [27], the aliphatic index of this protein was 65.33, and the high aliphatic index indicates the heat resistance of the protein [28].

**Table 2:** Physical and chemical parameters of lipase

Physical and chemical parameters	Parameter value
Amino acid residue number	240
Molecular mass	27,419.43 Da
Molecular formula	C_1199_H_1875_N_341_O_387_S_5_
Total number of positively charged residues	38
Total number of negatively charged residues	42
Total number of atoms	3807
Theoretical isoelectric point	5.44
Total average hydrophilicity value	-0.897
Fat index	65.33
Instability index	39.63

The amino acid composition of the protein is shown in Table S3, with aspartic acid (Asp), lysine (Lys), and alanine (Ala) accounting for the largest, 11.2%, 9.1%, and 8.3%, respectively. In contrast, cysteine (Cys) and histidine (His) accounting for the least, only 0.4% and 0.8%, respectively. The hydrophilicity and hydrophobicity of the protein were predicted by ProtScale software (https://web.expasy.org/protscale/), the results are shown in Fig. S1B. The number of negative peaks is more than the number of positive peaks, indicating that the protein is hydrophilic [29]. The secondary structure of the protein was predicted by using SOPMA (http://npsa-pbil.ibcp.fr/cgi-bin/npsa_automat.pl?page=npsa_sopma.html), and the results are shown in Fig. S1C, 129 amino acids were *α*-helix (53.75%), 12 amino acids were *β*-folded (5.00%), 27 amino acids were extended chain (11.25%) and 72 amino acids were irregularly coiled (30.00%).

### Homology modeling and molecular docking studie

**Fig. 6:**
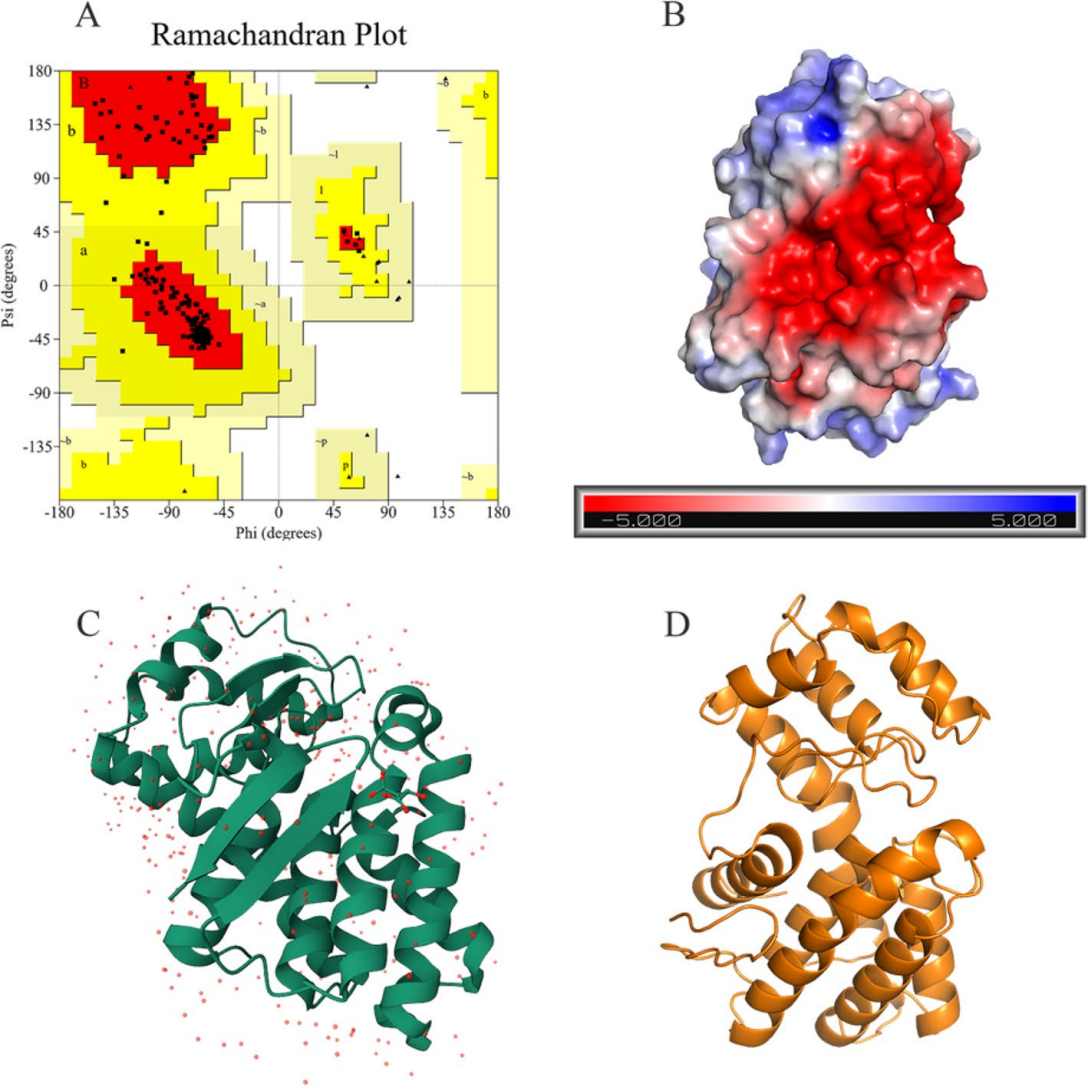
Laplace analysis and tertiary structure of lipase homology model. **A** The Ramachandran plot (red, yellow, light brown and white areas represent reliable, permissible,generous permissible and impermissible areas, respectively); **B** Electrostatic surface of purified lipase (blue represents positive, red represents negative); **C** Computational structure model of protein; **D** Crystal structure of protein

The Rasch analysis plot of the protein homology model is shown in Fig. 6A, with 203 residues in the reliable range (95.3%) and 10 residues in the permissive range (4.7%), and all residues in the reliable and permissive range. Verify 3D plots are shown in Fig. S3, 99.16% of residues have a mean 3D/1D score ≥ 0.1. The *α*-helix depression exists in the structure. The protein electrostatic surface is shown in Fig. 6B. The tertiary structure of the protein was predicted by SWISS-MODEL (https://swissmodel.expasy.org/interactive/), and its computational structural model and crystal structure after purification are shown in Fig. 6C and D.

**Fig. 7:**
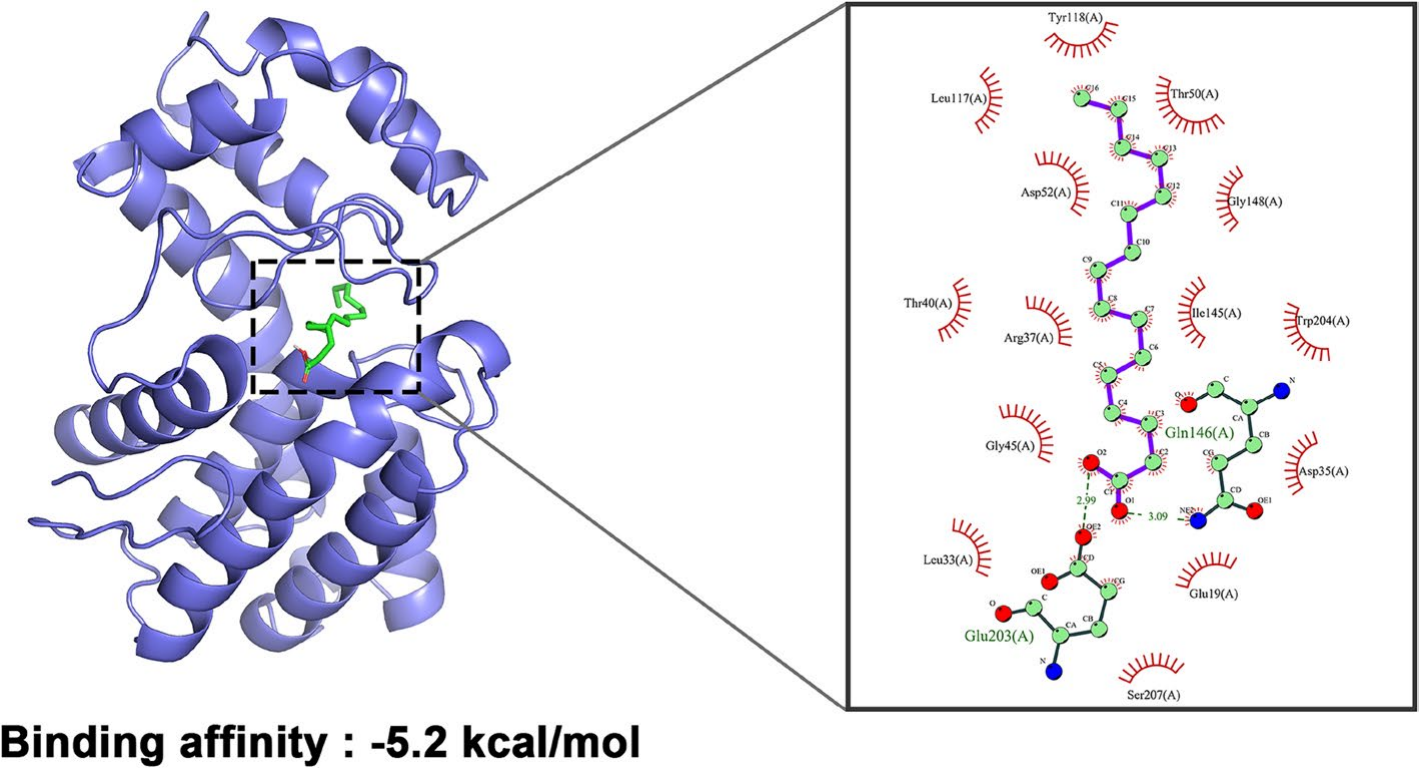
Docking model of lipase and palmitic acid (green represents hydrogen bonding)

The palmitic acid was selected in the Pubchem database (https://pubchem.ncbi.nlm.nih.gov/) for molecular docking with protein models in the Molecular Operating Environment (Autodock vina 12.0) platform, and the results are shown in Table 3 and Fig. 7. The results show that the ligand can bind to one structure (composed of multiple α-helix structures) of the protein. The affinity energy of palmitic acid for the model protein was -5.2 kcal/mol. It has been shown that proteins and molecules can spontaneously bind at affinity energies less than 0 kcal/mol, and the smaller the affinity energy value, the stronger the binding ability [30]. Palmitic acid binds to the protein via 2 hydrogen bonds at the Gln146(A) and Glu203(A) with bond lengths of 3.09 and 2.99, respectively. Follow-up work will be performed to mutate this locus for validation.

**Table 3:** Docking Results of Lipase and Palmitic Acid

Receptor protein	Ligand molecule	Binding energy (kcal/mol)	Hydrophobic binding site	Hydrogen bonding sites	Binding type
ZF2-lipase	Palmitic acid	-5.2	Tyr118(A) Leu117(A) Thr50(A) Asp52(A) Gly148(A) Thr40(A) Arg37(A) Ile145(A) Trp204(A) Gly45(A) Leu33(A) Asp35(A) Glu19(A) Ser207(A)	Gln146(A) Glu203(A)	Hydrophobic interaction

### Enzymatic properties study

#### Influence of temperature on lipase activity and stability

**Fig. 8:**
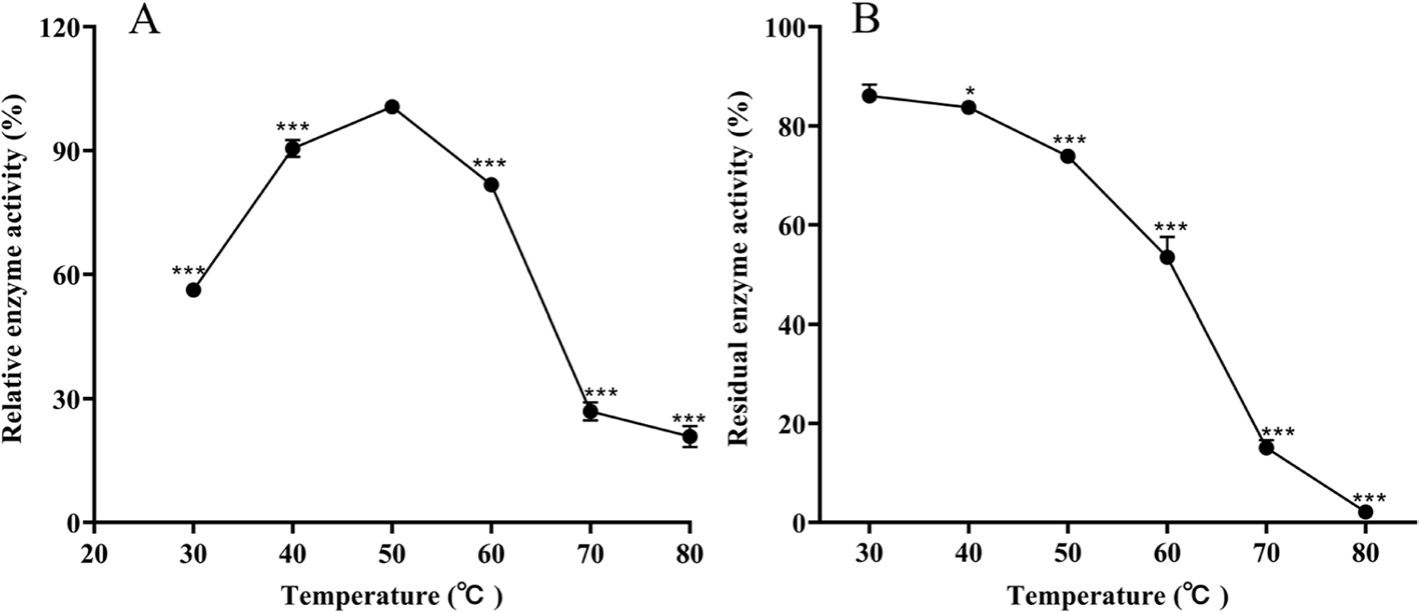
Changes of lipase at different temperatures (**A**) activity (**B**) residual activity

The effect of temperature on the activity and stability of lipase is shown in Fig. 8. With the increase of temperature, the relative enzyme activity showed a trend of increasing first and then decreasing (Fig. 8A), reaching a maximum value (100.64%) when the temperature reached 50 ℃. The lipase activity was stable at 30 to 50 °C, which was more than 70% (Fig. 8B). The enzyme activity gradually decreased with increasing temperature and was inactivated at 80 ℃.

The results indicate that lipase is a thermally active enzyme with good thermal stability. During the composting of pig carcasses, the generated biothermal heat can cause the pile to reach a high temperature of ≥ 55 ℃ and maintain the high temperature period. At this time, microorganisms begin to die or enter a dormant state, and extracellular enzymes produced by microorganisms can continue to work [31]. High temperatures are also often applied in many chemical reactions, promoting the dissolution of substrates and reducing the risk of microbial contamination [32]. This demonstrates that high temperature resistant enzymes have better application prospects.

### Effect of metal ions on lipase activity

In general, the expression of lipase activity is free from cofactors, but some specific metal ions can activate or inhibit the lipase activity [33]. The effects of different metal ions on lipase activity are shown in Table 4, and the results show that metal ions affected the activity of lipase. One mM Ag^+^ and Zn^2+^ promoted the enzyme activity, indicating that metal ions activated the lipase, possibly due to the metal ions changing the enzyme conformation, thus increasing the enzyme activity [34]. Similarly, 1 mM Cu^2+^, K^+^, and Fe^2+^; 5 mM Mn^2+^, Zn^2+^, Li^+^, Cu^2+^, K^+^, and Fe^2+^ completely inhibited enzyme activity. Under the remaining ions, lipase can maintain enzymatic activity, this indicates that lipase is resistant to metal ions.

**Table 4:** Changes in enzyme activity under different metal ions

Metal ion	Relative enzyme activity at different concentrations (%)	
	**1Mm**	**5mM**
Ag^+^	150 ± 1.24	16.67 ± 1.67
Mg^2+^	50 ± 1.32	100 ± 3.91
Ca^2+^	83.33 ± 2.12	66.67 ± 1.75
Mn^2+^	100 ± 2.6	0
Fe^3+^	83.33 ± 1.1	50 ± 1.45
Zn^2+^	133.33 ± 2.89	0
Li^+^	16.67 ± 3.15	0
Co^+^	33.33 ± 0.66	33.35 ± 1.6
Cu^2+^	0	0
K^+^	0	0
Fe^2+^	0	0

In previous studies, Ca^2+^ could increase lipase activity and it could also modulate the ligand structure to improve the thermal stability of the enzyme [35]. However, this study found that both 1 mM and 5 mM Ca^2+^ inhibited the lipase activity, this may be related to the protein structure of the lipase.

### Effect of organic solvents on lipase activity

Maintaining stability in organic solvents is one of the important characteristics of lipases. The effect of organic solvents on lipase activity is shown in Table 5. 5% isopropanol and toluene; 10% ethanol and methanol completely inhibited enzyme activity. 5% n-hexane; 10% isopropanol and n-hexane promoted the enzyme activity to 167%, 133%, and 150%, this may be due to organic solvent keeping the lipase in an open state, thus increasing the enzyme activity [36]. Under the remaining solvents, lipase can maintain enzymatic activity, the tolerance of lipases to organic solvents is crucial in industrial applications [37]. In this study, lipase was able to maintain activity in organic solvents, indicating that the enzyme has good prospects for application.

**Table 5:** Changes of enzyme activity under different organic solvents

Organic solvents	Relative enzyme activity at different concentrations (%)	
	**5%**	**10%**
Ethanol	66.67 ± 1.16	0
Isopropanol	0	133.33 ± 3.91
DMSO	100 ± 3.32	50 ± 1.64
Methanol	50 ± 1.2	0
N-hexane	167 ± 1.6	150 ± 2.26
Toluene	0	16.67 ± 1.45

### Effect of surfactants on lipase activity

Surfactants can reduce the tension at the water–oil interface [38], while increasing the contact surface at the water–oil interface [39]. The effects of different surfactants on lipase are shown in Table 6. Only 10% Tween 20 and Triton-x-100 completely inhibited enzymatic activity. Similar results were found in a previous study [40], possibly due to the structural alteration of the enzyme active center. Under the other surfactants, lipase maintained enzymatic activity, indicating that lipase can maintain enzymatic activity in the presence of surfactants. Overall, lipase can maintain enzymatic activity under different factors, this result lays the foundation for future applications.

**Table 6:** Changes of enzyme activity under different surfactants

Surface active agent	Relative enzyme activity at different concentrations (%)	
	**5%**	**10%**
SDS	16.67 ± 2.16	50 ± 2.26
Tween 20	83.33 ± 1.15	0
Tween 80	66.67 ± 3.21	33.33 ± 1.56
Gum Arabic	16.67 ± 1.16	33.33 ± 0.95
Triton-x-100	33.33 ± 2.1	0

### Degradation effect of lipase on kitchen oil

**Fig. 9:**
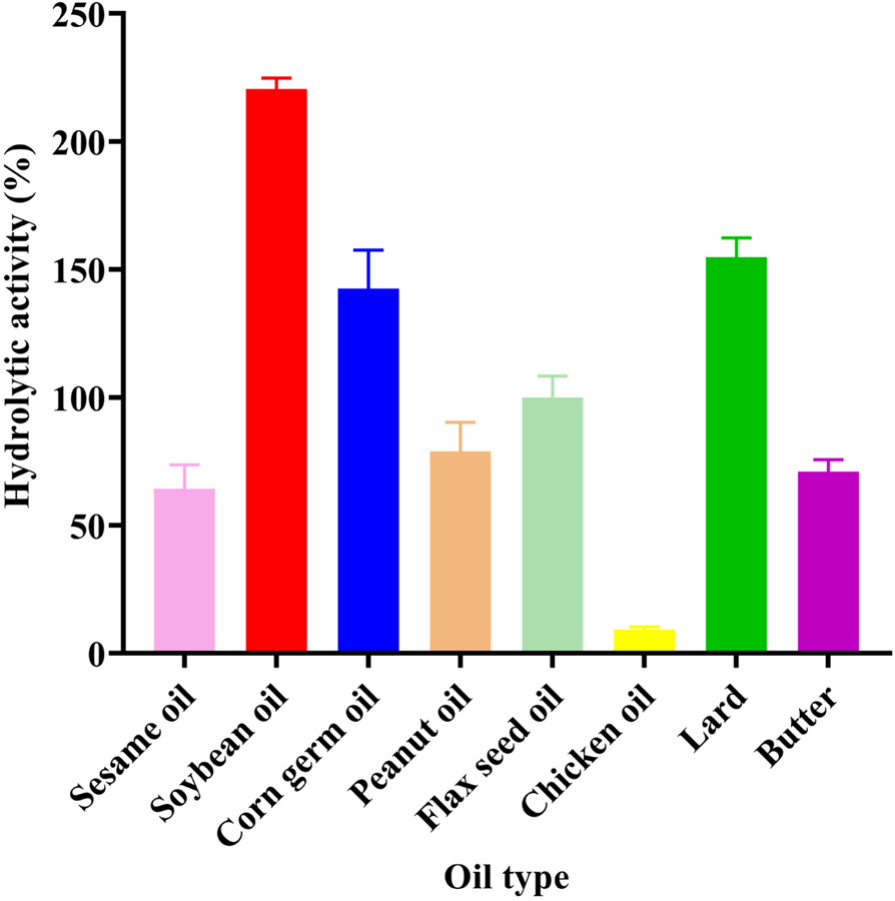
Enzymatic activity of lipase on different animal and plant fats

Used kitchen oil can cause damage to the environment, and waste oil can form an oily film on the surface of water bodies, thus interfering with the diffusion of oxygen [41]. To explore the enzymatic effect of ZF2 lipase on kitchen oil, enzymatic experiments were conducted by using kitchen oil as the substrate (Fig. 9). Among animal fats, the lowest enzyme activity was found in degrading chicken oil; the relative enzyme activity was 8.33% and the strongest in degrading lard; the relative enzyme activity was 158.2%. This may be related to the isolated source of the strain ZF2. Among vegetable fats, lipase showed the highest enzymatic activity in degrading soybean oil, with 222.223% relative activity. The lowest enzyme activity was found in degrading sesame oil, with relative activity reaching 64.3%. In this study, the lipase shows high enzymatic activity in degrading different plant and animal fats, demonstrating the potential of lipase to treat different fats. Its potential for degradation in more complex kitchen waste oils will be investigated subsequently.

## Conclusion

In this study, a lipase producing strain of *Bacillus* sp. ZF2 was isolated from the high temperature stage of pig carcass composting. Under the optimized conditions, the maximum lipase activity reached 32.46 U/mL. After two steps of purification, the specific activity of lipase reached 317.59 U/mg, a 9.78-fold elevation. lipase had the highest similarity to the GH family 46 chitosanase and molecular docking showed that lipase binds to fat via two hydrogen bonds at Gln146 (A) and Glu203 (A). The lipase was heat resistant and less affected by metal ions, organic solvents, and surfactants, and the lipase also showed degradation ability for different animal and vegetable fats. In short, lipase has the potential to degrade kitchen waste oil, future efforts will be devoted to the use of lipase with different enzyme preparations and the immobilization of lipase aiming to contribute to food and industrial waste treatment.

### Supplementary Information

**Additional file 1. **

## Data Availability

All data generated or analyzed during this study are included in this article and its supplementary information.
