# Supplementary material for: Preparation, purification, and biochemical of fat-degrading bacterial enzymes from pig carcass compost and its application
Source: BMC Biotechnol. 2023 Nov 3;23:48. doi: 10.1186/s12896-023-00818-1 (PMC10625193; doi:10.1186/s12896-023-00818-1)
Supplement: Supplementary file 1 — Additional file 1. [file 12896_2023_818_MOESM1_ESM.docx]

**Preparation, purification and biochemical of fat-degrading bacterial enzymes from pig carcass compost**

**and its application**

Xinran Duan^1^, Wei Zhai^1^, Xintian Li^1^, Sicheng Wu^1^, Ye Wang^1^, Lixia Wang^2^, Wangdui Basang^3^, Yanbin Zhu^3^, and Yunhang Gao^1*^

^1^College of Veterinary Medicine, Jilin Agricultural University, Changchun 130118, China

^2^Northeast Institute of Geography and Agroecology, Chinese Academy of Sciences, Changchun 130102, China

^3^Institute of Animal Husbandry and Veterinary Medicine, Tibet Academy of Agricultural and Animal Husbandry Science, Lhasa 850009, China

^*^Correspondence: gaoyunhang@163.com; Tel.: +86-131-5975-2912

**Table S1** Results of the initial screening of glycerol tributyrate medium. **(A)** ZF1; **(B)** ZF2; **(C)** DQ1; **(D)** DQ2

| **Strain** | **Ratio** |
| --- | --- |
| ZF1 | 1.52+0.09 |
| ZF2 | 1.69±0.26 |
| DQ1 | 1.14±0.16 |
| DQ2 | 0.99±0.12 |

*Ratio: ratio of hydrolysis circle diameter to strain diameter.

**Table S2** Lipase Amino Acid Sequence Statistics

| **Amino acid types** | **Amino acids contained** | **Number of amino acids** | **Percentage (%)** |
| --- | --- | --- | --- |
| Aliphatic | G, A,V, L,I | 76 | 31.67% |
| Aromatic | F, W,Y | 23 | 9.58% |
| Sulphur | C, M | 5 | 2.08% |
| Basic | K, R,H | 40 | 16.67% |
| Acidic | D, E,N, Q | 63 | 26.25% |
| Aliphatic hydroxyl | S, T | 26 | 10.83% |

**Table S3** Amino acid composition of lipase

| **Amino acid** | **Quantity** | **Proportion (%)** |
| --- | --- | --- |
| Ala（A） | 20 | 8.3 % |
| Arg（R） | 16 | 6.6 % |
| Asn（N） | 13 | 5.4 % |
| Asp（D） | 27 | 11.2 % |
| Cys（C） | 1 | 0.4 % |
| Gln（Q） | 9 | 3.7 % |
| Glu（E） | 15 | 6.2 % |
| Gly（G） | 18 | 7.4 % |
| His（H） | 2 | 0.8 % |
| Ile（I） | 7 | 2.9 % |
| Leu（L） | 18 | 7.4 % |
| Lys（K） | 22 | 9.1 % |
| Met（M） | 4 | 1.7 % |
| Phe（F） | 8 | 3.3 % |
| Pro（P） | 7 | 2.9 % |
| Ser（S） | 12 | 5.0 % |
| Thr（T） | 14 | 5.8 % |
| Trp（W） | 3 | 1.2 % |
| Tyr（Y） | 12 | 5.0 % |
| Val（V） | 14 | 5.8 % |

**
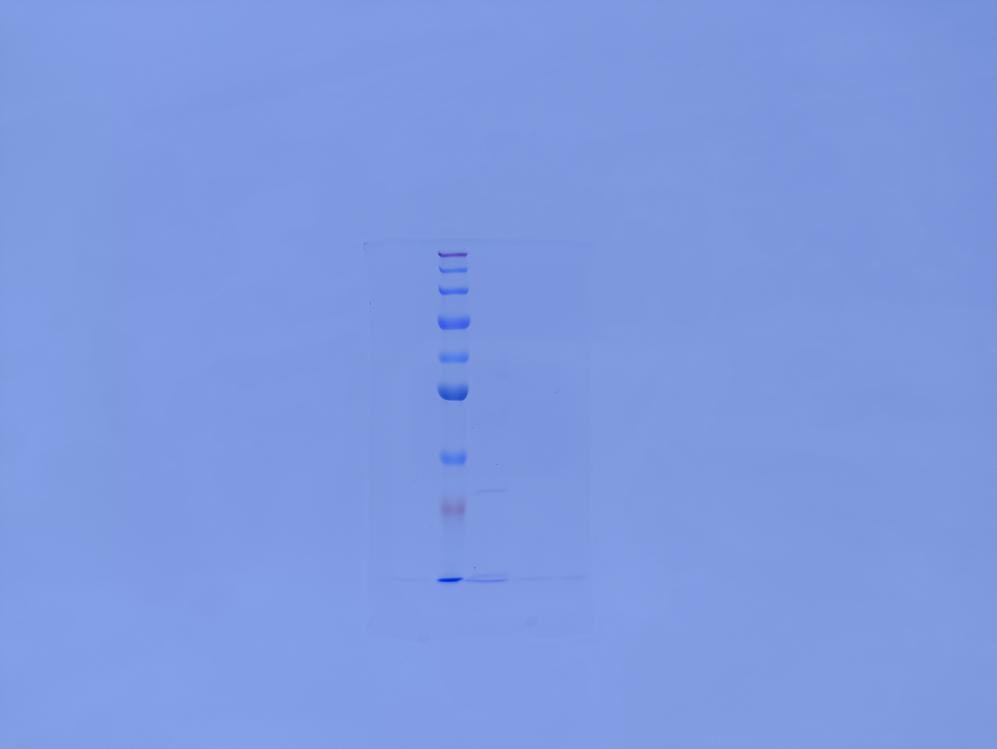

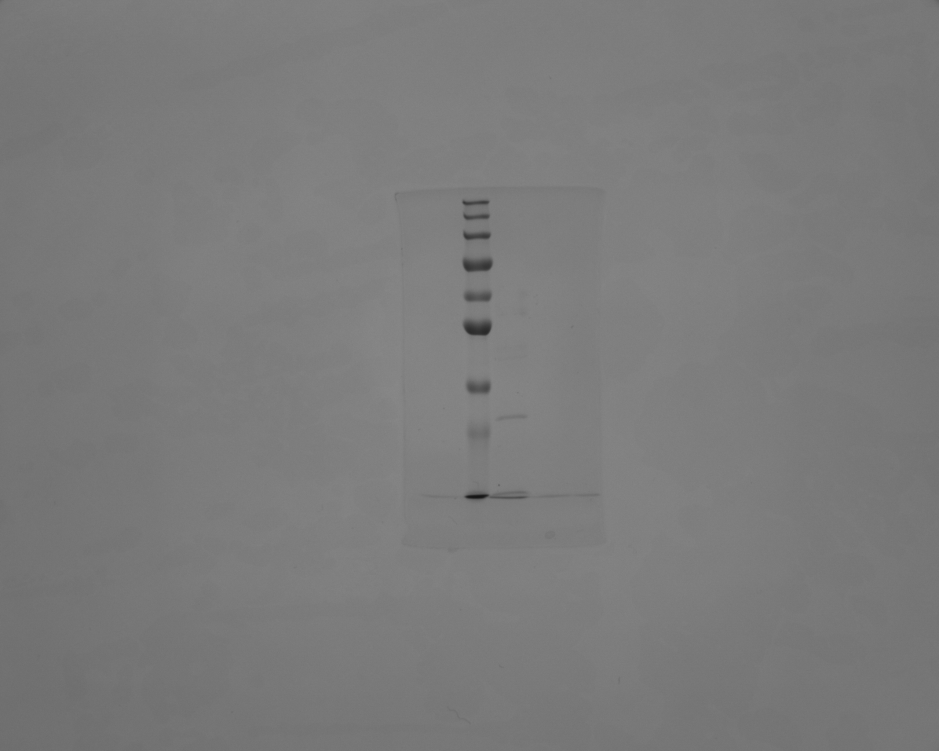
**

kDa

30 →

37 →

52 →

66 →

95 →

130 →

175 →

270 →

30 →

37 →

52 →

66 →

95 →

130 →

175 →

kDa

270 →

**Fig. S1** SDS-PAGE results of proteases at various purification steps (original and unprocessed versions).

The two charts are captured in different ways, but have the same content. From left to right: Marker; Sephadex G-75 purified lipase

**
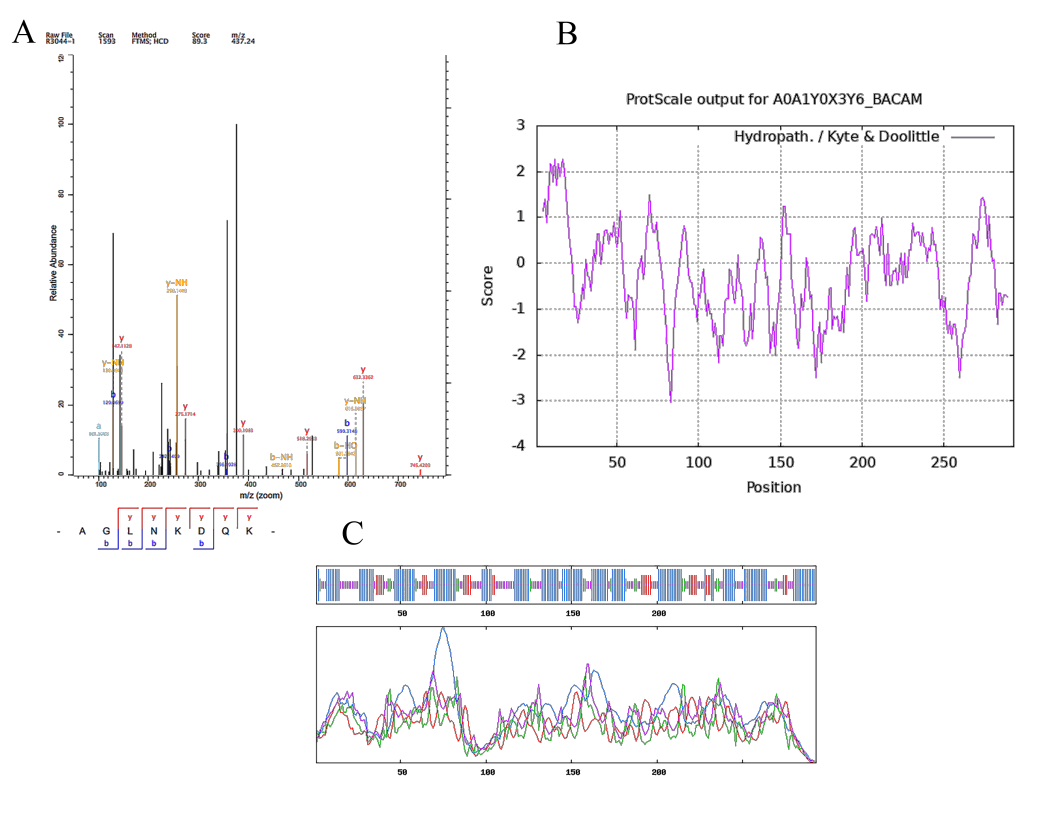
**

**Fig. S2** Secondary mass spectra **(A)**, hydrophilic analysis **(B)** and secondary structures **(C)**, blue for α-helix, red for extended chain, green for β-fold, yellow for irregular coiling) of purified lipase

**
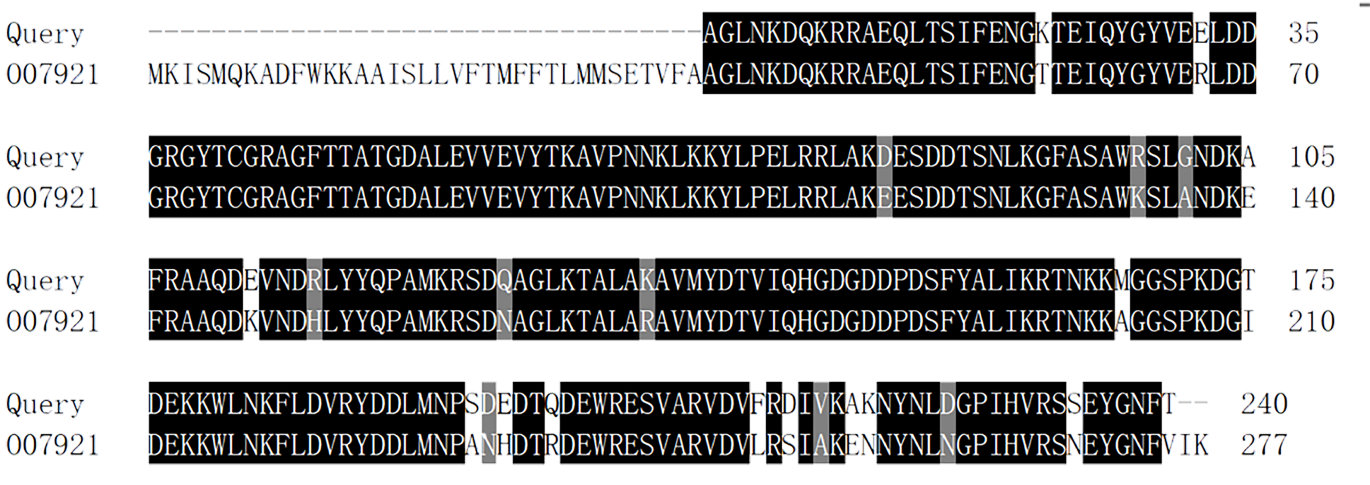
**

**Fig. S3** Homology analysis of purified lipase and O07921 amino acid sequences


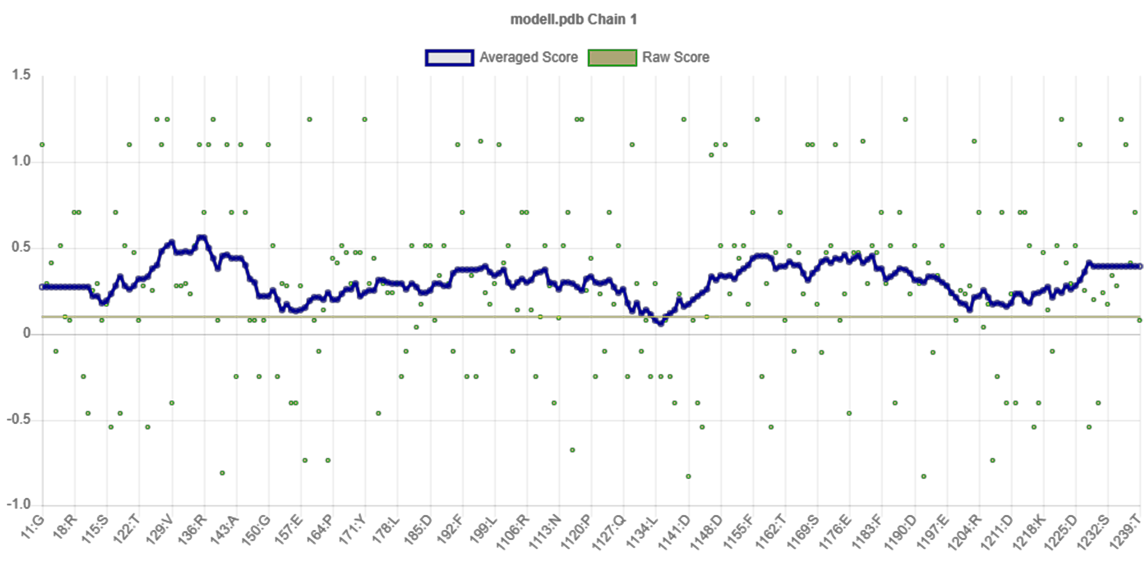


**Fig. S4** Receptor protein and small molecule docking scoring results
